# Some Encouraging Experiences

**Published:** 1916-07-22

**Authors:** 


### SOME ENCOURAGING EXPERIENCES.

The recent events in Northern France have gone far to justify the confidence which the country generally has never failed to feel in the ultimate issue of the European struggle. Not the least significant feature of the favourable development has been the steadiness with which it has been received. There has been no disposition to exaggerate the gain or to attach to it a premature or excessive interpretation. Just as quietness and patience and resolution have marked the period of waiting, so the same qualities have greeted the active signs of commencing triumph. Such a mood is in harmony with our best national traditions, and contradicts once more the prophecies which proclaimed us a decadent people. Both to our losses and to our gains we are able to bring the steadiness of the equal mind, and in quietness shall be our strength. If one point, more than others, in the general situation makes a universal appeal, it is the efficiency and personal valour of the new armies. The public has accepted this almost as a foregone conclusion, and with true British phlegm, but it has obviously impressed the professional soldier and military critic. The sacrifice of young lives is indeed sad to contemplate, but there is a quiet conviction that these have not been given for nought, and that the enterprise which involved them was directed with skill and sound judgment. The personal heroism of the soldier has not been spent in a vain show.

There are other evidences of national efficiency which speak in terms of encouragement and confidence. Our people at home are not merely waiting quietly, they are working, and this with energy and good will. That unceasing flow of munitions of war across the narrow seas speaks of men and women who are resolved that nothing shall fail in a great national enterprise, and who to this end bring both effort and sacrifice. Cherished regulations, trade rules, rightful holidays have been yielded in order that the claim for individual service shall not be made in vain; and the testimony is practically universal that all classes are united both in will and in action that, as we see the issue, freedom shall not perish from the earth. Possibly it may be suggested that this point of determination might well have been reached at an earlier date, but this, at best, is a barren controversy, and in any event the needed and expressive firmness is now attained. There is the consideration, also, that much which is now evident and declared is the fruit of long labours and preparations conducted hitherto in secret and out of the general view. Many other such activities, too, are still proceeding, to come to open display in due season. Here we may offer a word of encouragement to many members of the medical profession who have found that the call of national duty for them is to stay at home and to meet the extra burden of work which is entailed by the absence of colleagues summoned to more dramatic enterprises. In the care of the health of munition workers, and indeed of the civil population generally, they are contributing in no small measure to the end on which all are engaged, and by such duties they are filling a worthy part in the service of the nation. This may be obscure, but it is none the less real and substantial.

